# Ginsenosides Rg1 and Rg2 Activate Autophagy and Attenuate Oxidative Stress in Neuroblastoma Cells Overexpressing Aβ(1-42)

**DOI:** 10.3390/antiox13030310

**Published:** 2024-03-01

**Authors:** Ziqi Liu, Valentina Cecarini, Massimiliano Cuccioloni, Laura Bonfili, Chunmei Gong, Mauro Angeletti, Anna Maria Eleuteri

**Affiliations:** 1 School of Biosciences and Veterinary Medicine, University of Camerino, Via Gentile III da Varano, 62032 Camerino, Italy; ziqi.liu@unicam.it (Z.L.); massimiliano.cuccioloni@unicam.it (M.C.); laura.bonfili@unicam.it (L.B.); chunmei.gong@unicam.it (C.G.); mauro.angeletti@unicam.it (M.A.); annamaria.eleuteri@unicam.it (A.M.E.); 2 College of Food Science and Engineering, Jilin Agricultural University, Changchun 130118, China

**Keywords:** ginsenoside Rg1, ginsenoside Rg2, Alzheimer’s disease, autophagy, oxidative stress

## Abstract

Alzheimer’s disease is a neurodegeneration with protein deposits, altered proteolysis, and inflammatory and oxidative processes as major hallmarks. Despite the continuous search for potential therapeutic treatments, no cure is available to date. The use of natural molecules as adjuvants in the treatment of Alzheimer’s disease is a very promising strategy. In this regard, ginsenosides from ginseng root show a variety of biological effects. Here, we dissected the role of ginsenosides Rg1 and Rg2 in modulating autophagy and oxidative stress in neuroblastoma cells overexpressing Aβ(1-42). Key hallmarks of these cellular processes were detected through immunomethods and fluorometric assays. Our findings indicate that ginsenosides are able to upregulate autophagy in neuronal cells as demonstrated by increased levels of LC3II and Beclin-1 proteins and decreased amounts of p62. Simultaneously, an activation of lysosomal hydrolases was observed. Furthermore, autophagy activation promoted the clearance of Aβ(1-42). Rg1 and Rg2 also reduced oxidative stress sources and macromolecule oxidation, promoting NRF2 nuclear translocation and the expression of antioxidant enzymes. Our data further clarify the mechanisms of action of Rg1 and Rg2, indicating new insights into their role in the management of disorders like Alzheimer’s disease.

## 1. Introduction

Alzheimer’s disease (AD) is a neurodegenerative condition that burdens millions of people in the world, mostly the elderly. A huge body of studies have shown that amyloid-β (Aβ) deposition into plaques, hyperphosphorylated tau proteins, altered proteolysis, oxidative stress, and neuroinflammation are associated with the onset and progression of AD [1]. Despite the continuous search for potential therapeutic treatments, no cure for AD is available to date. Among the different approaches, targeting AD-associated oxidative stress emerged as one of the most promising strategies. Briefly, oxidative stress occurs when the amount and effects of ROS/RNS become overwhelming for the antioxidant system, with this event leading to neuronal cell damage [2]. Analyses of AD brains have demonstrated a great impact of oxidative damage, suggesting oxidative stress as a central event in the pathogenesis of the disorder [3].

AD is also characterized by proteostasis failure with dysfunctional proteasome and autophagy. Autophagy is considered the major pathway for the clearance of damaged organelles and aggregated and long-lived proteins and is essential for neuronal homeostasis and the balance of energy and nutrients [4]. Increasing evidence has demonstrated the presence of defective autophagy in several neurodegenerative diseases, particularly AD [5,6]. Thus, therapeutic strategies aimed to enhance autophagy may be beneficial in this pathology.

Autophagy and oxidative stress are intimately correlated in cells. The activation of autophagy is a critical event in the cellular response to oxidative stress, as it removes defective components before further damage/aggregation occurs [7]. Autophagy counteracts the increase in ROS amount, either promoting the clearance of compromised mitochondria via mitophagy or degrading KEAP1 and favoring the release and activation of NRF2 and the subsequent expression of the antioxidant response element (ARE) region that regulates drug metabolism and disposition, antioxidant defense, and oxidant signaling [8,9,10].

Ginseng is a traditional herb that has been used in Eastern countries for more than 5000 years in a variety of traditional medical therapies, and it represents an interesting and promising source of compounds with drug-like properties. Ginsenosides are triterpene saponins and constitute the main active ingredients in ginseng. They show several functional properties, including antioxidant, anti-inflammatory, anticancer, and immunomodulatory functions [11,12,13,14]. Among the different ginsenosides, Rg1 and Rg2 are protopanaxatriol (PPT) type ginsenosides, which have been shown to possess also anticoagulant, antiapoptotic, antioxidant and antidiabetic properties. Previous studies have demonstrated the ability of these compounds to exert protective effects on neurodegenerative diseases including Parkinson’s disease and AD. Rg1 is the most abundant and active ginsenoside in ginseng root. It was shown to regulate oxidative stress, apoptosis, and neuroinflammation through the Wnt/GSK-3β/β-Catenin signaling pathway thereby improving cognition in AD [15]. Additionally, ginsenoside Rg1 exerted neuroprotective mechanisms and ameliorated memory deficits in 5XFAD mouse models restoring PINK-Parkin-mediated mitophagy [16]. As for ginsenoside Rg2, Cui et al. showed that it may improve hippocampal neuronal damage and reduce Aβ(25-35)-induced memory impairment in rats by increasing the PI3K/Akt signaling pathway [17]. Furthermore, it was demonstrated to alleviate neurovascular damage in 3xTg-AD mice through the MAPK-ERK pathway [18].

In this study, we further explored the mechanisms of action of ginsenosides Rg1 and Rg2, evaluating their ability to modulate autophagy and oxidative stress in human neuroblastoma SH-SY5Y cells stably transfected with either the wild-type amyloid precursor protein gene (APPwt) or the 717 valine-to-glycine AβPP-mutated gene (APPmut). This mutation associates with familial forms of AD and favors the accumulation of amyloid-β peptides in the brain resulting in the enhancement of amyloid fibrils and making these cells an appropriate model to explore the events occurring in this neurodegeneration. We previously detected APP levels in the two clones showing that they express comparable amount of the protein but higher than those observed in control cells [19]. Additionally, these cells released significantly higher amounts of Aβ(1-42) than non-transfected cells [20,21]. Here, we demonstrate the ability of both ginsenosides to upregulate autophagy and lower oxidative stress in neuronal cells overexpressing Aβ(1-42), thus confirming their cytoprotective function.

## 2. Materials and Methods

### 2.1. Regents and Chemicals

Materials for cell cultures, including media, reagents, and plastics, were purchased from Corning (Tewksbury, MA, USA). Ginsenosides Rg1 (purity ≥ 98%, C_42_H_72_O_14_, molecular weight 801.03, CAS No 22427-39-0) and Rg2 (purity ≥ 97%, C_42_H_72_O_13_, molecular weight 785.03, CAS No 52286-74-5) were purchased from Extrasynthese (Genay, France). The reagent 3-(4,5-Dimethyl-2-thiazolyl)-2,5-diphenyl-2H-tetrazolium bromide (MTT) used in cell viability assays was purchased from Merck Spa (Milan, Italy). Membranes for Western blotting analyses were obtained from Millipore (Milan, Italy). For the immunodetection, the enhanced chemiluminescence (ECL) system (Amersham Pharmacia Biotech, Milan, Italy) was used. Primary antibodies used in Western blotting assays to monitor p62, Beclin-1, LC3, 3-NT, 4-HNE, 8-oxodG, NRF2, Lamin B1, GST, HO-1, OGG1, and GAPDH were obtained from Santa Cruz Biotechnology (Heidelberg, Germany).

### 2.2. Cell Treatment and Cell Viability Assay

SH-SY5Y cells were cultured in Dulbecco’s modified Eagle’s medium (DMEM)/Nutrient Mixture F12 (1:1) containing 10% fetal bovine serum (FBS), 2 mM glutamine, 100 units/mL penicillin, and 100 μg/mL streptomycin, incubated at 37 °C in an environment containing 5% CO_2_. APPwt and APPmut clones were a kind gift of Prof. Daniela Uberti from the University of Brescia. Cells were stably transfected as described in [19] and maintained in medium supplemented with G418 600 μg/mL. Cells were treated for 24 h with 50 μM of Rg1 and Rg2 (both dissolved in DMSO). Control cells were treated with same amount of DMSO and experiments were performed in triplicate. The morphology of control and treated cells was observed under an inverted light microscope (Leica DM IL, Leica Microsystems, Wetzlar, Germany) at 40× magnification. Cell viability studies were conducted with the MTT assay. Briefly, cells were incubated with increasing concentrations of Rg1 and Rg2 (0–500 μM) for 24 h and washed with cold phosphate-buffered saline (PBS, pH 7.5), then 0.5 mg/mL MTT was added to the medium without FBS and incubated at 37 °C for 4 h. The medium was then replaced with 100 μL of DMSO, and the optical density was measured at 550 nm in a microtiter plate reader. Three independent experiments, each with six replicates, were performed.

### 2.3. Cathepsin Activities

Cathepsin B and L proteolytic activities were measured in cell lysates as previously described [22] using the fluorogenic peptides Z-Arg-Arg-AMC and Z-Phe-Arg-AFC, respectively (5 μM final concentration). For cathepsin B activity, 7 μg of protein lysate were pre-incubated in 100 mM phosphate buffer pH 6.0, 1 mM EDTA, and 2 mM dithiothreitol for 5 min at 30 °C. After substrate addition, the solution was incubated for 15 min at 30 °C. For cathepsin L activity, 7 μg of protein lysate were incubated in 100 mM sodium acetate buffer pH 5.5, 1 mM EDTA, and 2 mM dithiothreitol for 5 min at 30 °C, and, upon substrate addition, the solution was incubated for 15 min at 30 °C. The fluorescence corresponding to the hydrolyzed 7-amino-4-methyl-coumarin (AMC, λ_exc_ = 365 nm, λ_em_ = 449 nm) and 7-amino-4-trifluoromethylcoumarin (AFC, λ_exc_ = 397 nm, λ_em_ = 500 nm) was detected on a SpectraMax Gemini XPS microplate reader (Molecular Devices, San Jose, CA, USA). The real cathepsin contribution to substrate degradation was evaluated by performing control activity assays with the specific inhibitor CA074Me and subtracting these values from those obtained analyzing cell lysates.

### 2.4. Molecular Docking

The molecular models of complexes formed between ginsenosides Rg1 and Rg2 and human cathepsin B were obtained according to flexible ligand–receptor docking using Autodock 4 software [23]. The chemical structures of the molecules of interest were retrieved from Pubchem, 3D-optimized with Avogadro [24], and docked onto the crystallographic structure of human cathepsin B and cathepsin L (PDB ID: 1CSB [25] and PDB ID: 3HHA [26]), which was obtained from the RCBS Protein Data Bank. Briefly, a grid box (25 × 25 × 25 Å) was placed around the putative allosteric regulating region of cathepsin B (the site being identified by analogy with the established allosteric activating site present on cathepsin Z (PDB ID: 5JA7) [27]), the box being centered on the Trp-166 residue and covering the entire surface extending 12.5 Å in each direction. Unless stated differently, default settings were applied throughout. Resulting models were analyzed using Maestro (Schrödinger Release 2023-4: Maestro, Schrödinger, LLC, New York, NY, USA, 2023) and rendered with PyMOL (The PyMOL Molecular Graphics System, Version 2.4 Schrödinger, LLC).

### 2.5. Western Blotting

After 24 h exposure to ginsenosides, the medium was removed from the plates and cells were washed and collected in 4 mL of PBS and centrifuged at 1600× *g* for 5 min. Pellets were resuspended in a lysis buffer (20 mM Tris, pH 7.4, 250 mM sucrose, 1 mM EDTA and 5 mM β-mercaptoethanol) and passed through a 29-gauge needle at least ten times. Lysates were centrifuged at 12,000× *g* for 15 min and the supernatants were stored at −80 °C until use. For NRF2 determination, cytosolic and nuclear proteins were collected after treatments. Protein concentration in lysates was determined using the Bradford assay [28]. Proteins were separated by 12–15% SDS-PAGE and transferred onto PVDF membranes. Membranes were then incubated with primary monoclonal antibodies and successively with the specific peroxidase-conjugated secondary monoclonal antibodies (Abcam, Cambridge, UK). Proteins were detected at the ChemiDoc MP system (Bio-Rad, Segrate (MI), Italy). Gels were loaded with molecular weight markers (12–225 kDa, GE Healthcare, Munich, Germany). Membranes were stripped (stripping buffer containing 200 mM glycine, 0.1% SDS, and 1% Tween 20) and tested with an anti-GAPDH or an anti-Lamin B1 monoclonal antibody to verify for equal protein loading. The ImageJ 1.52a software (NIH, Bethesda, MD, USA) was used to quantify protein bands that were normalized to GAPDH or Lamin B1.

### 2.6. Oxyblot Analysis

The content in protein carbonyl groups was measured with The Oxyblot Protein Oxidation Detection kit. Briefly, 7 μg of protein lysates were incubated with 2,4-dinitrophenylhydrazine (DNPH) at room temperature to generate 2,4-dinitrophenylhydrazone (DNP-hydrazone). The DNPH-derivatized samples were then separated by SDS-PAGE and transferred to the PVDF membrane. The membrane was incubated with an anti-DNP antibody and a specific secondary antibody, both provided by the kit. The ECL system was utilized for the detection. Staining with Coomassie Brilliant Blue was performed to check for protein loading.

### 2.7. ROS Production and Mitochondrial Membrane Potential

To determine ROS generation, the DCFDA (dichlorodihydrofluorescein diacetate)—Cellular ROS Assay Kit (Abcam, ab113851) was used. Control and transfected SH-SY5Y cells were cultured in a black and flat bottom 96-well microplate and exposed to 50 µM Rg1 and Rg2 for 24 h. Cells were then incubated with 20 µM DCFDA for 45 min at 37 °C in the dark. Finally, the fluorescence intensity was detected at excitation and emission wavelengths of 485 nm and 535 nm using a fluorescence microplate reader (SpectraMax Gemini XPS, Molecular Devices, San Jose, CA, USA).

JC-1 Mitochondrial membrane potential assay kit (Abcam, ab113850, Cambridge, UK) was used to determine Mitochondrial Membrane Potential (MMP). Cells were seeded at 30,000 cells/well in a black and flat bottom 96-well microplate and incubated with 50 µM Rg1 and Rg2 for 24 h. Then, the cells were incubated with 10 µM of JC-1 dye for 20 min. After washing with PBS, the quantification of relative fluorescence intensity was measured using a fluorescence microplate reader (SpectraMax Gemini XPS, Molecular Devices, San Jose, CA, USA). The red fluorescence intensity, corresponding to the aggregate form of JC-1, was measured using excitation and emission wavelengths of 535 and 590 nm, respectively, and the green fluorescence intensity, corresponding to the monomers of JC-1, was measured using excitation and emission wavelengths of 490 and 530 nm.

### 2.8. Quantification of Aβ(1-42) in Cell Medium and Cell Lysates

The amounts of the peptide Aβ(1-42) present in cell extracts and secreted into the medium after exposure to Rg1 and Rg2 were measured with the Human Aβ42 ELISA Kit from Invitrogen (Carlsbad, CA, USA), following manufacturer’s instructions. Cell culture medium collected after treatment was centrifuged at 300× *g* for 10 min to remove non-adherent cells and debris, and finally protease inhibitors were added.

### 2.9. Statistical Analysis

Data are expressed as mean values ± S.D. Statistical analysis was performed with one-way ANOVA, followed by the Bonferroni post hoc test using Sigma-stat 3.1 software (SPSS, Chicago, IL, USA), and *p* < 0.01 or *p* < 0.05 were considered statistically significant.

## 3. Results

### 3.1. Effect of Rg1 and Rg2 on Cell Viability

**Figure 1:**
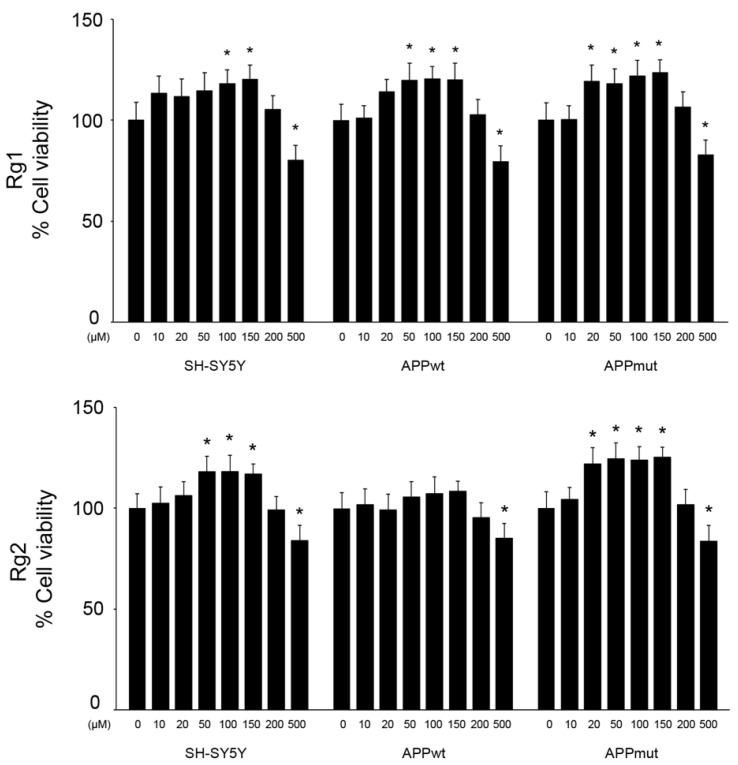
SH-SY5Y cell viability upon Rg1 and Rg2 treatment. Control and transfected SH-SY5Y cells were treated with 0–500 μM of the ginsenosides Rg1 and Rg2 and cell viability was assessed with the MTT assay. Results are expressed as percentages compared to DMSO-treated cells. Three independent experiments, each with six replicates, were performed. Asterisks indicate data points statistically significant compared to the respective DMSO-treated control (* *p* < 0.05).

Firstly, we aimed at evaluating the effects of the two ginsenosides on the viability of control and transfected SH-SY5Y cells. The MTT assay was used to test a range of concentrations of the two compounds (from 0 up to 500 μM) and cells were visualized under a bright field microscope to investigate eventual morphological changes (Figure 1 and Supplementary Figure S1). Interestingly, both ginsenosides induced a slight but significant increase in cell viability with concentrations in the range of 50 μM–150 μM. Specifically, a nearly 20% increase in cell viability was observed in the three cell lines exposed to the ginseng metabolites, with the only exception being APPwt cells treated with Rg2, where no change in viability was detected. A moderate decline in cell viability was measured upon exposure to 500 μM of Rg1 and Rg2. No morphological changes were observed for concentrations up to 200 μM, whereas rounded cells were found after treatment with 500 μM of both ginsenosides, in accordance with the reduced viability.

### 3.2. Effect of Rg1 and Rg2 on Autophagy-Related Proteins

**Figure 2:**
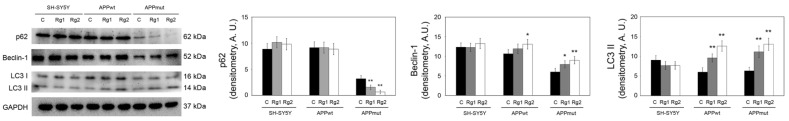
Expression of autophagy-related proteins in control and transfected SH-SY5Y cells upon 24 h treatment with Rg1 and Rg2. Representative immunoblots and densitometric analyses obtained from five independent experiments are shown (A.U., arbitrary units). GADPH staining was used to check for equal protein loading. Asterisks indicate data points statistically significant compared to the respective DMSO-treated cells (* *p* < 0.05, ** *p* < 0.01).

Autophagy is considered a major cellular quality-control system able to maintain homeostasis, and numerous findings provided evidence for its deregulation in AD [29]. We investigated the role of the two ginsenosides in modulating autophagy functionality measuring the expression levels of key autophagic proteins; in detail, LC3, Beclin-1, and p62 (Figure 2). LC3 is the most widely used specific marker during autophagy initiation and is related to the number of autophagosomes. Its cytosolic form, LC3 I, is converted into the membrane-bound form LC3 II to initiate the formation of the autophagosome. Beclin-1 exerts its activity by forming a multimeric complex with other macromolecules. p62 can associate LC3 and ubiquitinated substrates and is subsequently integrated into autophagosomes and degraded in autophagolysosomes. Specifically, p62 is negatively correlated with autophagic activity, and reflects the intensity of autophagic degradation capacity and autophagic flux [30,31]. Our results show that Rg2 was the most active between the two tested ginsenosides with the highest effect on the abovementioned markers obtained in APPmut cells, whereas no change was detected in the control SH-SY5Y cells. In this clone, ginsenosides induced a complete activation of autophagy, with an increase in LC3 II and Beclin-1 levels, both involved in the initiation stage of autophagy, and a simultaneous significant downregulation of the p62 protein (2.0-fold and 5.0-fold decrease with Rg1 and Rg2, respectively), whose levels inversely correlate with autophagy activity. As for APPwt cells, treatments induced an upregulation of LC3 II and Beclin-1 levels but no differences in the amount of p62 were detected, indicating the activation of the initial steps of this degradation process.

**Figure 3:**
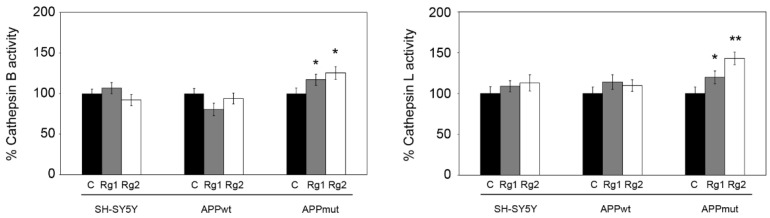
Cathepsin B and L activities detected in control and transfected SH-SY5Y cells after 24 h treatment with 50 µM Rg1 and Rg2. Activity assays were performed using fluorogenic peptides as substrates as described in the Section 2. Data are indicated as percentages versus DMSO-treated control/transfected cells (* *p* < 0.05, ** *p* < 0.01).

**Figure 4:**
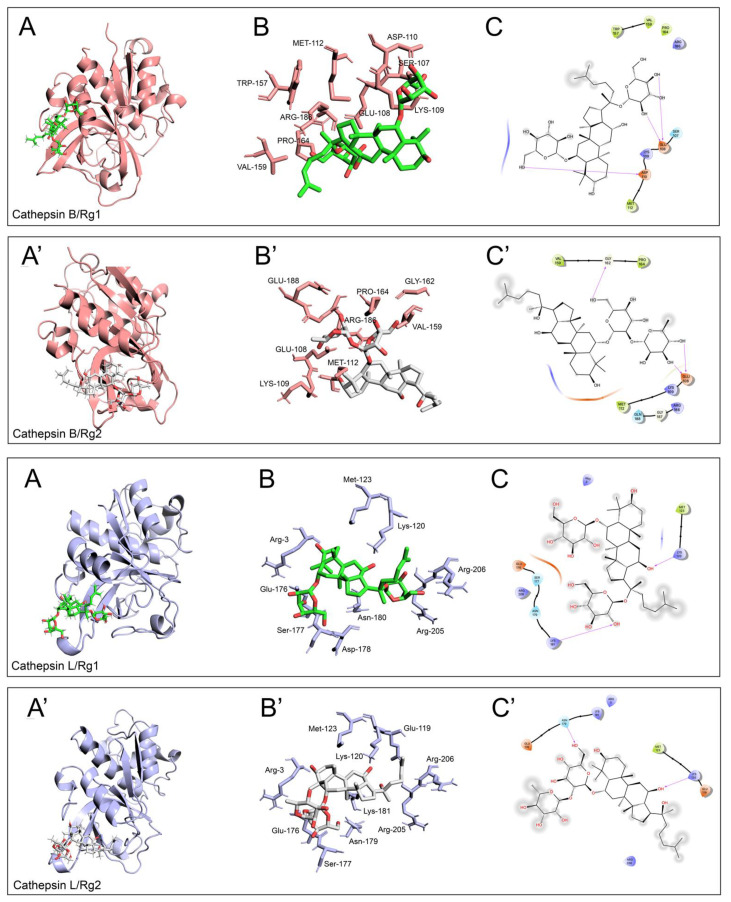
Visualization of computed binding modes of Rg1 and Rg2 to human cathepsin B (**upper** panels, the enzyme is indicated in pink, pdb ID: 1csb) and cathepsin L (**lower** panels, the enzyme is indicated in light blue, pdb ID: 3HHA). Best-scoring models of the complexes obtained upon docking onto human cathepsin B and L of ginsenoside Rg1 and Rg2 (panels (**A**) and (**A′**), respectively). Detailed 3D visualizations of amino acids constituting the allosteric site of cathepsin B and L and directly involved in the binding are reported in panels (**B**,**B′**); 2D visualization of the residues directly involved in the formation of H-bonds (purple arrows), polar (light blue ribbon), and directly involved in the formation of H-bonds (purple arrows), polar (light blue ribbon), and VdW interactions (green ribbon) are shown in panels (**C**,**C′**). Grey circles indicate atoms/groups exposed to solvent.

We then monitored the functionality of cathepsin B and L, the most abundant among all lysosomal hydrolases [32], with a role in AD pathogenesis and progression. In APPmut cells, we obtained an activation of both enzymes, mainly evident for cathepsin L (20% and 40% activation in the presence of Rg1 and Rg2, respectively) (Figure 3). A less marked increase was obtained for cathepsin B that showed a 15% and 22% activation upon exposure to Rg1 and Rg2, respectively. Supported by previous studies exploring the regulating role of ginsenoside on the activity of cellular enzymes [33], we hypothesized that the observed increase in cathepsin activity could involve also a direct allosteric mechanism stimulated by the binding of Rg1 and Rg2 to the L subdomain of the enzyme (Figure 4) [34]. As for the SH-SY5Y control and APPwt cells, no changes in cathepsin activity were detected upon treatments.

### 3.3. Determination of Aβ(1-42) in Cell Lysates and Cell Medium

**Figure 5:**
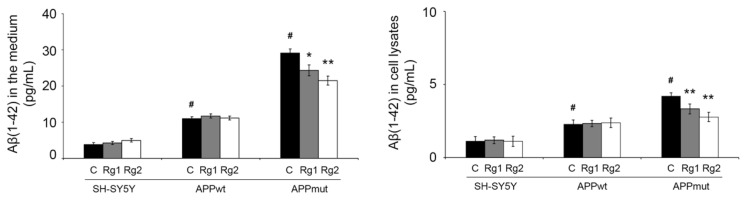
Levels of Aβ(1-42) peptides in cell medium and extracts upon 24 h exposure to 50 µM ginsenosides Rg1 and Rg2. A commercial ELISA kit was used, and data are indicated as percentages vs. DMSO-treated control/transfected cells (* *p* < 0.05, ** *p* < 0.01). # indicates significant differences in terms of amyloid production and release between control and transfected cells (*p* < 0.01).

The levels of the peptide Aβ(1-42) were determined in the medium and in cellular extracts of control and APPwt/APPmut-transfected SH-SY5Y cells treated with Rg1 and Rg2. It was previously demonstrated that APPmut cells produced and released higher amounts of Aβ(1-42) peptide than APPwt cells [20,21]. Here, we prove that ginsenosides, particularly Rg2, significantly reduced the levels of Aβ(1-42) both in the medium and in cell extracts (Figure 5). No change in the levels of the peptide were observed in non-transfected and APPwt cells.

### 3.4. ROS Production and Mitochondrial Membrane Potential

**Figure 6:**
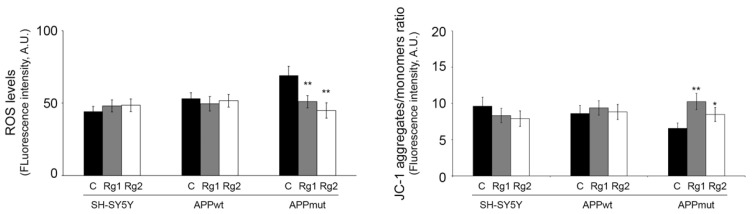
ROS levels (DCFDA) and mitochondrial membrane potential (JC-1) in control and transfected SH-SY5Y cells after 24 h treatment with 50 µM Rg1 and Rg2. Cells were stained with 20 µM DCFDA and 10 µM JC-1 and then the fluorescence emission was evaluated as reported in the Section 2. Fluorescence intensities of the dyes are presented as arbitrary units (A.U.). (* *p* < 0.05, ** *p* < 0.01).

Next, we investigated the effect of ginsenosides on intracellular ROS accumulation and mitochondrial membrane potential (MMP), which are well-known mediators of oxidative stress. ROS levels were detected with the fluorescent compound DCF. Data shown in Figure 5 indicate that in the absence of treatments, APPmut cells release higher amounts of ROS compared to control SH-SY5Y cells, confirming the high levels of oxidative stress previously described for this clone [19]. ROS levels in APPmut cells were decreased by approximately 20% by treatment with Rg1/Rg2 (Figure 6, left panel). As for untransfected and APPwt cells, no differences were obtained in ROS production. MMP was measured using the fluorescent dye JC-1 that accumulates in the mitochondrial matrix of healthy cells in the form of aggregates and produces a red fluorescence. A decrease in MMP blocks JC-1 accumulation in the matrix and favors its existence as a monomer, producing green fluorescence. The ratio of red/green (aggregates/monomers) is used as an indicator of variations in MMP. Considering untreated cells, the APPmut clone shows decreased MMP compared to control untreated SH-SY5Y cells, which indicates the presence of damaged mitochondria (Figure 6, right panel). Interestingly, treatment with ginsenosides restores MMP in APPmut cells. Again, no change was evidenced for both control and APPwt-transfected SH-SY5Y cells.

### 3.5. Oxidation of Cellular Macromolecules

**Figure 7:**
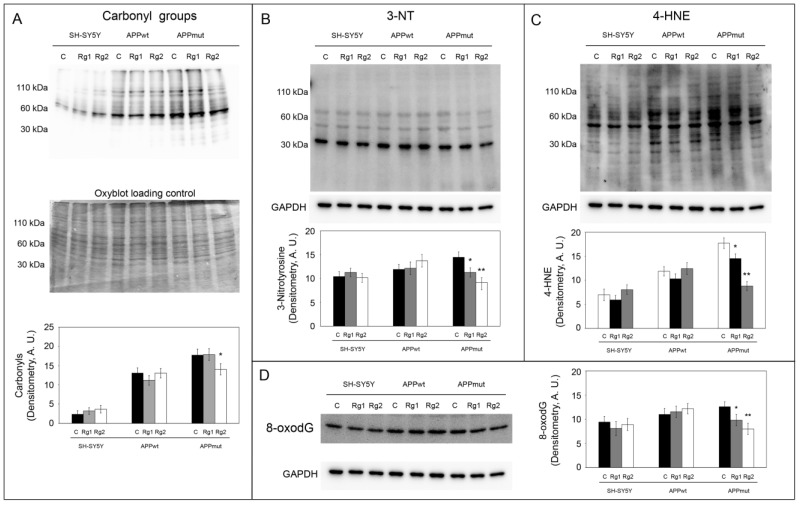
Determination of protein carbonyls (**A**), 3-nitrotyrosines (**B**), lipid 4-HNE adducts (**C**) and 8-oxodG (**D**) in control and treated cells. Representative immunoblots and densitometric analyses from five independent experiments are shown (A.U., arbitrary units). Values are expressed as mean ± standard deviation. Data are indicated as percentages versus DMSO-treated control/transfected cells (* *p* < 0.05, ** *p* < 0.01). Equal protein loading for carbonyl groups was checked, staining the membrane with Coomassie after the Oxyblot procedure, (**A**) whereas for 3-NT, 4-HNE adducts, and 8-oxodG, it was verified by using an anti-GAPDH antibody.

To monitor the antioxidant potential of the two ginsenosides, we detected some markers of macromolecules’ oxidation (Figure 7). Higher levels of all the considered markers were observed in APPmut cells compared to untransfected cells, confirming previous data on the altered redox state of this clone [19]. Carbonyl groups and 3-NT are known as important hallmarks of protein oxidation. In APPmut cells, a 1.26-fold and 1.6-fold reduction for carbonyls and 3-NT was observed upon exposure to Rg2, whereas Rg1 only diminished the levels of 3-NT (1.38-fold reduction) (Figure 7A,B). The protein bound 4-HNE was measured to highlight lipid peroxidation products. Both ginsenosides diminished the levels of 4-HNE with Rg1 being the most effective (2.0-fold reduction) (Figure 7C). DNA oxidation levels were monitored through the expression of the DNA oxidation product 8-oxodG (Figure 7D). As observed for the previously described markers, Rg1 and Rg2 decreased the amount of 8-oxodG in APPmut cells, inducing a 1.56- and a 1.28-fold reduction, respectively. No changes in the levels of the considered markers were observed in control and APPwt-transfected cells.

### 3.6. Upregulation of Antioxidant Enzyme Expression

**Figure 8:**
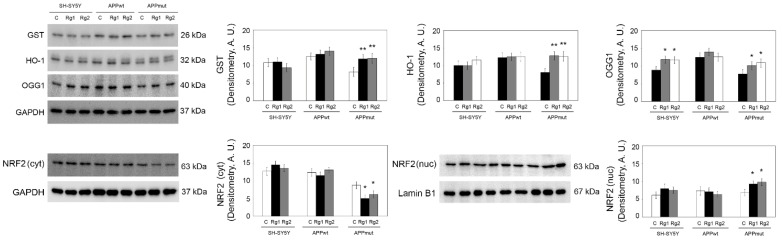
Determination of the expression levels of HO-1, GST, OGG1, and NRF2 in control and treated cells. NRF2 was detected in both cytosolic and nuclear extracts. Representative immunoblots and densitometric analyses obtained from five independent experiments are shown (A.U., arbitrary units). Values are expressed as mean ± standard deviation. Data are indicated as percentages versus DMSO-treated control/transfected cells (* *p* < 0.05, ** *p* < 0.01). Equal protein loading was verified by using an anti-GAPDH antibody (cytosolic proteins) or an anti-Lamin B1 antibody (nuclear fraction).

To further explore the antioxidant ability of both Rg1 and Rg2, we evaluated the expression of antioxidant enzymes. The enzyme heme oxygenase-1 (HO-1) shows strong anti-inflammatory and antioxidant properties. It mediates the conversion of heme into biliverdin, with the release of free iron and carbon monoxide. Biliverdin is then rapidly metabolized to bilirubin, a potent antioxidant [35]. Glutathione transferase (GST) is involved in detoxification processes and is able to provide protection against aldehydic by-products of lipid peroxidation, including 4-hydroxynonenal HNE [36]. We also monitored the expression of 8-oxoguanine DNA glycosylase-1 (OGG1) that detoxifies oxidized guanine base lesions produced by free radicals. APPmut cells treated with the ginsenosides showed an upregulation of these enzymes, particularly evident for the HO-1 which showed a 1.59- and 1.56-fold increase with Rg1 and Rg2, respectively (Figure 8). OGG1 resulted upregulated upon treatments also in untransfected SH-SY5Y cells. No effects were obtained on APPwt cells.

These enzymes are under the control of NRF2, a transcriptional factor master regulator of cellular responses to oxidative, inflammatory, and metabolic stresses [37]. We investigated the expression levels of this protein upon neuronal cells’ treatment with Rg1 and Rg2. In line with the findings on the upregulation of antioxidant enzymes, we obtained an activation and nuclear translocation of NRF2 demonstrated by decreased levels of the protein in the cytosol and increased amounts in nuclear fractions of APPmut cells treated with ginsenosides, compared to control not-treated cells (Figure 8).

## 4. Discussion

The present study dissects the role of ginsenosides Rg1 and Rg2 in regulating autophagy and oxidative stress in neuronal cells overexpressing Aβ(1-42). AD is considered the most common type of dementia and is described as a progressive multifactorial neurodegenerative pathology characterized by neuronal cell death, protein deposits, altered proteolysis and redox homeostasis. During the past few years, research has increased the understanding of AD mechanisms and pathogenesis, but current treatments can only help to control some symptoms. Furthermore, approved drugs such as donepezil, rivastigmine and galantamine can alter the course of the disease but they show some negative side effects [38]. Recently, the use of natural bioactive compounds to prevent or slow down the course of the disorder has become an interesting new topic for researchers. Ginsenosides from ginseng represent a class of natural molecules showing interesting biological activities. The root or stem of ginseng have been used for centuries in many Asian countries to treat a variety of diseases. Based on their chemical structure, ginsenosides are grouped into four types, the protopanaxadiol-, protopanaxatriol-, ocotillol- and oleanolic acid-types. Several studies have already suggested ginseng and ginsenosides’ promising roles as adjuvant treatments to existing medications in AD [39,40,41]. 

In the present work, we dissected the ability of the ginsenosides Rg1 and Rg2, which are in the 20(S)-protopanaxatriol category [12], to modulate autophagy and oxidative stress in APPwt and APPmut cells. These two clones express higher amounts of APP and Aβ(1-42) than non-transfected cells [19,20,21], thus they can be considered consistent models to investigate molecular events occurring in AD.

Autophagy and oxidative stress are both directly involved in AD but their relationship and accurate mechanisms in neurodegenerative processes still require further investigation. Defects in the autophagic pathway have been reported to mediate neurotoxicity and favor AD. In particular, the abnormal degradation and consequent accumulation of autophagosomes in neurons is a key event in the development of this disorder [42]. Considering its role in the degradation of misfolded/aggregated proteins and in the clearance of damaged organelles, including mitochondria, the correct functionality of autophagy is fundamental for cell survival since it prevents protein deposits and limits the production of ROS [42,43]. Here, we demonstrate that ginsenosides Rg1 and Rg2 can modulate the autophagic machinery in APPmut cells as proved by the increased expression of LC3 II and Beclin-1, both involved in autophagosome formation, and the reduced levels of p62, which inversely correlate with autophagy. In APPwt cells, treatments only initiate early autophagy events as indicated by higher levels of LC3 and Beclin-1 and no changes in p62. These data also denote that the final effect of ginsenosides on autophagy depends on the expression of the wild-type or mutated sequence of the APP. In line with our results, Fan et al. found that the ginsenoside Rg2 increased the clearance of aggregated proteins by inducing autophagy and enhanced cognitive function in AD mice [44].

It is known that the final degradation of autophagic substrates occurs in lysosomes by the means of hydrolases such as cathepsins B and L. In our experiments, the activity of both enzymes, mainly cathepsin L, was increased in APPmut cells treated with Rg1 and Rg2, confirming the complete activation of autophagy by ginsenosides in this clone. To explain the observed activation, considering previous studies on ginsenosides and their ability to regulate the activity of cellular enzymes [33], it is reasonable to hypothesize the involvement of a direct allosteric mechanism induced by the binding of Rg1 and Rg2 to the L subdomain of the enzyme [34]. The role of cathepsins in AD has been deeply investigated by numerous research groups, and controversial data has emerged [32]. Since cathepsins can be found in other cellular locations besides lysosomes, they can also exert non-canonical roles. Many cathepsin substrates are proteins associated with neurodegeneration, such as APP, Aβ, α-synuclein, huntingtin, and prion proteins [32]. The exact function of cathepsin B in AD and in APP processing remains to be defined. Some authors suggested its involvement in APP cleavage and Aβ generation, exacerbating neuronal deficits in AD [45], on the other hand, other studies indicated its anti-amyloidogenic role with the ability to degrade Aβ peptides, resulting in neuroprotection [46,47]. As for cathepsin L, it can upregulate α-secretase and thereby suppress Aβ levels [48]. To better clarify the role of activated autophagy, we detected the amounts of Aβ(1-42) in cell lysates and in the medium after treatments. Ginsenosides significantly reduced the concentration of the peptide in both samples, indicating that the upregulation of autophagy favors the degradation of this toxic peptide.

Among the different roles, autophagy plays an important function in removing damaged mitochondria which are a major intracellular source of ROS and oxidative stress. Considering the ability of Rg1 and Rg2 to activate autophagy in AD-like cells, we investigated markers of oxidative stress in these cell lines. We observed that APPmut cells treated with Rg1 and Rg2 showed an ameliorated oxidative profile with reduced amounts of ROS and improved MMP. Additionally, all the tested markers of oxidative damage to macromolecules (4-HNE, 3-NT, carbonyls and 8-oxodeoxyguanosine) resulted downregulated in the same cellular clone. In a previous paper, we dissected the oxidative condition of APPmut cells, showing that 4-HNE, 3-NT, and carbonyls levels were all significantly higher in comparison to APPwt cells and SH-SY5Y non-transfected cells. Moreover, the levels of mitochondrial ROS produced in APPmut cells were higher compared to non-transfected cells [19]. Here, we demonstrate that Rg1 and Rg2 significantly reduce oxidative stress markers in APPmut cells to levels comparable to those found in control cells and in the APPwt clone.

Redox homeostasis is a complex condition with a balance between oxidative stress sources and antioxidant defense systems. We analyzed a panel of antioxidant enzymes playing a role in the detoxification of the previously described oxidative markers; in detail, HO-1, GST, and OGG1. HO-1 is a cytoprotective enzyme able to reduce oxidative stress and to act against the toxicity of protofibrillar Aβ(1-42) through the inhibition of AMPK activation [49]. GST protects against the harmful by-products of lipid peroxidation [36]. OGG1 is the enzyme responsible for the excision of 8-oxoguanine. The expression of the three enzymes was increased in APPmut cells upon ginsenosides’ treatment, further contributing to ameliorate the oxidative profile of these cells and in line the above-described reduction of macromolecules’ oxidation products. The induction of the expression of these enzymes was also confirmed by the nuclear translocation of NRF2, a key regulator of antioxidant and detoxifying genes’ transcription.

Interestingly, considering that HO-1 promotes cell survival by diminishing oxidative stress and maintaining mitochondrial integrity [50,51], it could be also argued that ginsenosides act by reducing oxidative stress via HO-1 upregulation and the consequent amelioration of mitochondrial functionality.

Concluding, our results suggest a novel mechanism of action for ginsenosides Rg1 and Rg2 indicating their beneficial and protective role in cells through the activation of autophagy, the consequent clearance of the Aβ(1-42) peptide, and the decrease in oxidative stress levels and support their role as adjuvants to conventional drugs to improve amyloidopathies’ treatment.

## Data Availability

The data presented in this study are available on request from the corresponding author.

## Supplementary Materials

The following supporting information can be downloaded at: https://www.mdpi.com/article/10.3390/antiox13030310/s1, Figure S1: Cytotoxicity of ginsenosides in control and untransfected SH-SY5Y cells. Microscopic images of SH-SY5Y cells after a 24 h treatment with Rg1 and Rg2 ginsenosides at 0, 200 and 500 μM.
