# Supplementary material for: Ginsenosides Rg1 and Rg2 Activate Autophagy and Attenuate Oxidative Stress in Neuroblastoma Cells Overexpressing Aβ(1-42)
Source: Antioxidants (Basel). 2024 Mar 1;13(3):310. doi: 10.3390/antiox13030310 (PMC10967604; doi:10.3390/antiox13030310)
Supplement: Supplementary file 1 [file antioxidants-13-00310-s001.zip › antioxidants-2834379-supplementary.pdf]

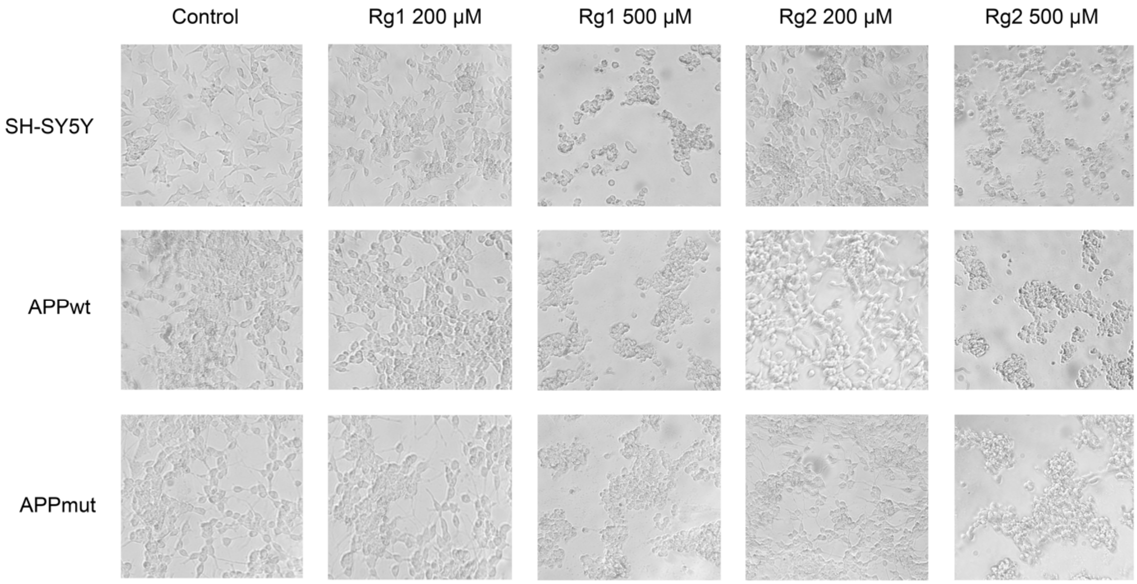

Figure S1. Cytotoxicity of ginsenosides in control and untransfected SH-SY5Y cells. Microscopic images of SH-SY5Y cells after a 24 h treatment with Rg1 and Rg2 ginsenosides at 0, 200 and 500 µM.
